# Angiopoietin-2 Promotes Inflammatory Activation in Monocytes of Systemic Sclerosis Patients

**DOI:** 10.3390/ijms21249544

**Published:** 2020-12-15

**Authors:** Tiago Carvalheiro, Ana P. Lopes, Maarten van der Kroef, Beatriz Malvar-Fernandez, Carlos Rafael-Vidal, Anneline C. Hinrichs, Nila H. Servaas, Femke Bonte-Mineur, Marc R. Kok, Lorenzo Beretta, Maili Zimmermann, Wioleta Marut, Jose M. Pego-Reigosa, Timothy R. D. J. Radstake, Samuel Garcia

**Affiliations:** 1 Center for Translational Immunology, University Medical Center Utrecht, University of Utrecht, 3508 GA Utrecht, The Netherlands; t.ferreiracarvalheiro@umcutrecht.nl (T.C.); A.P.PinheiroLopes-3@umcutrecht.nl (A.P.L.); m.vanderkroef@umcutrecht.nl (M.v.d.K.); beatrizmalvar@gmail.com (B.M.-F.); a.c.hinrichs-2@umcutrecht.nl (A.C.H.); N.H.Servaas-2@umcutrecht.nl (N.H.S.); m.zimmermann@umcutrecht.nl (M.Z.); w.k.marut@umcutrecht.nl (W.M.); tradstake73@gmail.com (T.R.D.J.R.); 2 Department of Rheumatology and Clinical Immunology, University Medical Center Utrecht, University of Utrecht, 3584 CX Utrecht, The Netherlands; 3 Rheumatology & Immuno-mediated Diseases Research Group (IRIDIS), Galicia Sur Health Research Institute (IIS Galicia Sur), SERGAS-UVIGO, 36312 Vigo, Spain; carlos.rafael@iisgaliciasur.es (C.R.-V.); jose.maria.pego.reigosa@sergas.es (J.M.P.-R.); 4 Rheumatology Department, University Hospital Complex of Vigo, 36313 Vigo, Spain; 5 Department of Rheumatology and Clinical Immunology, Maasstad Hospital Rotterdam, 3079 DZ Rotterdam, The Netherlands; bontef@maasstadziekenhuis.nl (F.B.-M.); kokmr@maasstadziekenhuis.nl (M.R.K.); 6 Referral Center for Systemic Autoimmune Diseases, Fondazione IRCCS Ca’ Granda Ospedale Maggiore Policlinico di Milano, 20122 Milan, Italy; lorberimm@hotmail.com

**Keywords:** angiopoietin-2, systemic sclerosis, monocyte activation, interleukin-6, interleukin-8

## Abstract

Angiopoietin-2 (Ang-2), a ligand of the tyrosine kinase receptor Tie2, is essential for vascular development and blood vessel stability and is also involved in monocyte activation. Here, we examined the role of Ang-2 on monocyte activation in patients with systemic sclerosis (SSc). Ang-2 levels were measured in serum and skin of healthy controls (HCs) and SSc patients by ELISA and array profiling, respectively. mRNA expression of *ANG2* was analyzed in monocytes, dermal fibroblasts, and human pulmonary arterial endothelial cells (HPAECs) by quantitative PCR. Monocytes were stimulated with Ang-2, or with serum from SSc patients in the presence of a Tie2 inhibitor or an anti-Ang2 neutralizing antibody. Interleukin (IL)-6 and IL-8 production was analyzed by ELISA. Ang-2 levels were elevated in the serum and skin of SSc patients compared to HCs. Importantly, serum Ang-2 levels correlated with clinical disease parameters, such as skin involvement. Lipopolysaccharide (LPS) LPS, R848, and interferon alpha2a (IFN-α) stimulation up-regulated the mRNA expression of *ANG2* in monocytes, dermal fibroblasts, and HPAECs. Finally, Ang-2 induced the production of IL-6 and IL-8 in monocytes of SSc patients, while the inhibition of Tie2 or the neutralization of Ang-2 reduced the production of both cytokines in HC monocytes stimulated with the serum of SSc patients. Therefore, Ang-2 induces inflammatory activation of SSc monocytes and neutralization of Ang-2 might be a promising therapeutic target in the treatment of SSc.

## 1. Introduction

Systemic sclerosis (SSc) is a rheumatic and musculoskeletal (RMD) disease characterized by excessive collagen deposition in the skin and internal organs. Besides the fibrotic phenotype, SSc is also characterized by vascular abnormalities and the activation of immune cells, including monocytes and macrophages [1,2,3,4]. Monocyte activation leads to the secretion of cytokines (interleukin (IL)-6, IL-8), chemokines (C-C motif ligand (CCL)-2, C-X-C motif ligand (CXCL)-10), and pro-fibrotic factors such as tissue inhibitor of matrix metalloproteinases (TIMP)-1, which are elevated in SSc patients and are involved in the inflammatory and fibrotic processes observed in the pathogenesis of the disease [2,3,5,6,7,8].

Ang-2, together with Ang-1, is the best-characterized ligand of Tie2, which is a tyrosine kinase receptor essential for vascular development and blood vessel stability [9]. Although Tie2 is mainly expressed by endothelial cells, several works have shown that it is also expressed in myeloid cells, such as monocytes, synovial macrophages, and monocyte-derived macrophages [10,11,12,13]. Recent works from our group have shown that Ang-2 mediated-Tie2 activation induces the production inflammatory mediators by rheumatoid arthritis (RA) and psoriatic arthritis (PsA) macrophages and that Ang-2 neutralization reduces the severity of a mouse model of arthritis [12,14], pointing out the role of Ang-2 in the pathogenesis of these RMDs.

An imbalance in the levels of Ang-2 has been also previously observed in SSc patients, as Ang-2 levels were increased in the serum of these patients compared to HCs [15,16,17,18,19]. In addition, increased Ang-2 levels have been associated with the severity of skin and lung involvement and vascular alterations in SSc [15,18,19]. However, the role of Ang-2 on monocyte activation in SSc pathology remains unknown.

## 2. Results

### 2.1. Ang-2 Levels Are Elevated in the Serum of SSc Patients

**Figure 1:**
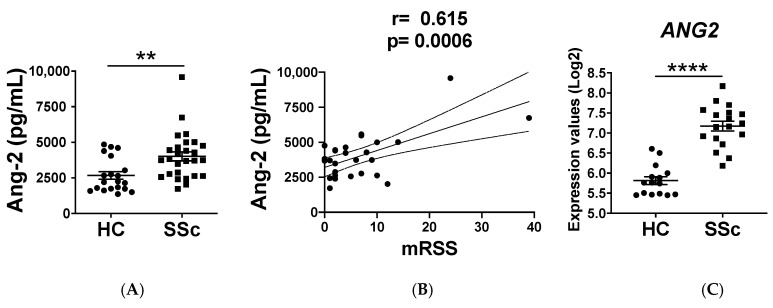
(**A**) Angiopoietin-2 (Ang-2) levels are elevated in systemic sclerosis (SSc) patients. Ang-2 levels in the serum of healthy controls (HCs) (*n* = 20) and SSc patients (*n* = 27). (**B**) Correlation of Ang-2 serum levels with modified Rodnan skin score (mRSS). (**C**) *ANG2* expression in the skin of HCs (*n* = 15) and affected skin of dcSSc patients (*n* = 18). Means and standard error mean (SEM) are shown. ** *p* < 0.01 and **** *p* < 0.0001.

We first analyzed the serum levels and Ang-2 in our cohort of patients. Similar to previous findings [15,16,17,18,19], Ang-2 levels were significantly elevated in SSc patients compared to HCs (Figure 1A). This dysregulation was found both in the limited cutaneous (lcSSc) and in the diffuse cutaneous (dcSSc) SSc patients (Figure S1) and, interestingly, Ang-2 levels positively correlated with the modified Rodnan skin score (Figure 1B). Due to the association of Ang-2 with the skin involvement, we next determined the Ang-2 expression levels in the affected skin of SSc patients. For this purpose, we made use of the available array profiling data from Mantero JC P. et al. (GSE95065) and we found that the *ANG2* mRNA expression was significantly higher in the affected skin of dcSSc patients compared to the skin of HCs (Figure 1C).

**Figure 2:**
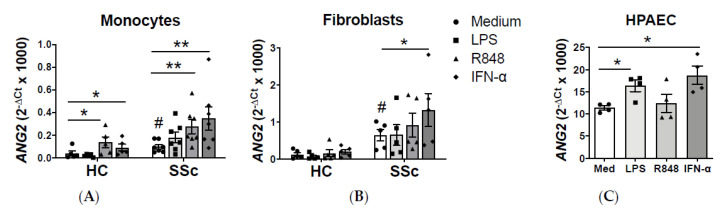
Ang-2 levels are elevated in monocytes and dermal fibroblast of SSc patients. (**A**–**C**) *ANG2* mRNA expression in monocytes (**A**) and dermal fibroblasts (**B**) from HC and SSc patients (*n* = 5–6) from HPAECs (**C**) stimulated with lipopolysaccharide (LPS) LPS, R848 or interferon alpha2a (IFN-α) for 24 h (*n* = 4). Means and SEM are shown. * *p* < 0.05 and ** *p* < 0.01. # *p* < 0.05 compared to HC medium.

Next, we sought to determine the cell types involved in the increased levels of Ang-2. We found that the basal levels of *ANG2* were higher in monocytes and fibroblast from SSc patients compared to HCs (Figure 2A,B). Furthermore, we investigated the effect of inflammatory mediators involved in the pathogenesis of SSc, such as toll-like receptor (TLR) agonists [2,3] and interferon- (IFN-) as SSc is a type-I IFN disease [20]. In monocytes, stimulation with LPS (TLR4 ligand) did not modulate the expression of *ANG2*, but R848 (TLR7/8 ligand) and IFN- stimulation significantly upregulated its expression in both HC and SSc patients (Figure 2A). On the dermal fibroblast only IFN- induced the expression of *ANG2* in SSc patients (Figure 2B). As endothelial cells are the main producers of Ang-2 [9], we also analyzed the effect of HPAEC stimulation and we found that LPS and IFN- significantly enhanced the expression of *ANG2* (Figure 2C). Together, these data showed an increase of Ang-2 levels in SSc patients, and that monocytes, dermal fibroblasts, and endothelial cells might be involved in this dysregulation.

### 2.2. Monocyte-Ang-2 Stimulation Induces IL-6 and IL-8 Secretion in SSc Patients

**Figure 3:**
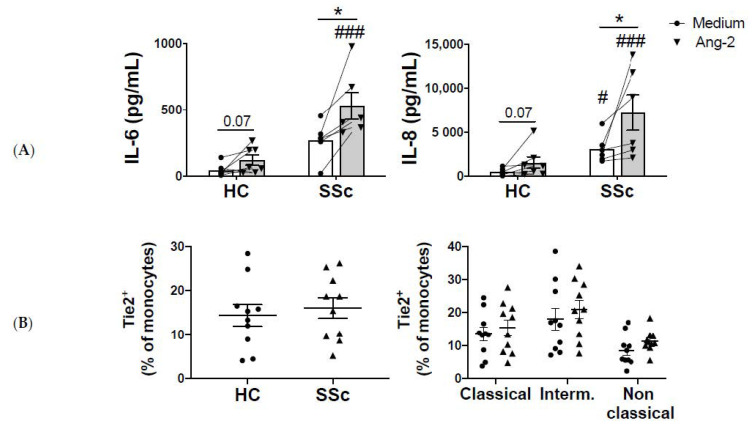
Ang-2 induces Interleukin (IL)-6 and IL-8 production in monocytes of SSc patients. (**A**) IL-6 and IL-8 production in HC and SSc monocytes (*n* = 8 each) after 24-h incubation in medium or Ang-2 (200 ng/mL). (**B**) Percentage of Tie2^+^ expressing monocytes and monocyte subsets in HC and SSc patients’ monocytes (*n* = 10 each). Means and SEM are shown. * *p* < 0.05, # *p* < 0.05 and ### *p* < 0.001 compared to medium.

Next, we analyzed the functional consequences of Tie2 activation in monocytes. The basal production of IL-6 and IL-8 was higher in monocytes of SSc patients compared to HC monocytes, although the differences were only significant in IL-8. In HC monocytes, Ang-2 stimulation showed a trend towards a higher production of IL-6 and IL-8. In SSc monocytes, Ang-2 induced a strong and significant production of both IL-6 and IL-8 (Figure 3A). We also determined the secretion of TIMP-1, as its expression is enhanced in monocytes of SSc patients [6]. However, Ang-2 did not regulate the expression of TIMP-1, neither in HC nor SSc patients (data not shown). In order to discard the possibility that the higher effect observed on SSc monocytes was due to a higher expression of Tie2, we analyzed its expression levels in monocytes of SSc patients and HCs. We did not observe differences in the percentage of Tie2^+^ cells, neither in total monocytes, nor in the different monocyte subsets (classical, intermediate, and non-classical, Figure 3B).

**Figure 4:**
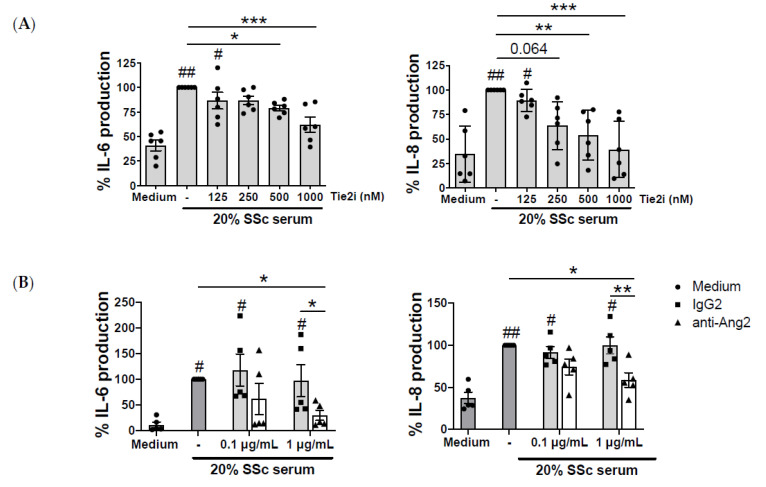
Tie2 inhibition and Ang-2 neutralization reduce the systemic sclerosis serum-induced monocyte IL-6 and IL-8 production. (**A**) IL-6 and IL-8 production by HC monocytes, pre-treated for 1 h with increasing concentrations of Tie2 inhibitor (Tie2i) and stimulated for 24 h with serum of SSc patients (20% *v*/*v*). Data are presented as a percentage of production relative to SSc serum from 5 independent experiments. (**B**) IL-6 and IL-8 production by HC monocytes after 24-h incubation with the serum of SSc patients (20% *v*/*v*), pre-treated for 1 h with an isotype control (IgG2) or an anti-Ang-2 neutralizing antibody. Data are presented as a percentage of production relative to SSc serum, from 6 independent experiments. Means and SEM are shown. * *p* < 0.05, ** *p* < 0.01 and *** *p* < 0.001. # *p* < 0.05 and ## *p* < 0.01 compared to medium.

These results suggest that Ang-2 contributes to the elevated IL-6 and IL-8 levels found in SSc patients. To validate this hypothesis, we analyzed the effect of Tie2 inhibition on the SSc serum-induced cytokine secretion by monocytes. The serum of SSc patients induced the secretion of both cytokines by HC monocytes and this secretion was reduced dose dependently by Tie2 inhibition (Figure 4A). Finally, to prove that this inhibition was Ang-2 dependent we neutralized Ang-2 in the serum of SSc patients and we found a significant reduction of IL-6 and IL-8 monocyte secretion (Figure 4B).

## 3. Discussion

Several works have previously shown that Ang-2 is elevated in SSc patients [15,16,17,18,19], but its role on the activation of SSc monocytes has not been investigated yet. In our cohort of SSc patients, and in line with the previous reports, the levels of Ang-2 were elevated compared to HCs. In vitro data showed that mRNA *ANG2* levels were higher in monocytes and dermal fibroblasts of SSc patients compared to HC. Moreover, TLR and IFN-α induced the expression of *ANG2*. These data suggest that both TLR signaling and IFN signature, which are important in the pathogenesis of SSc [3,20], are involved in the elevated Ang-2 expression observed in these patients.

Our results also demonstrated that Ang-2 induces the secretion of IL-6 and IL-8 by SSc monocytes. Similarly, this effect was also observed in HC monocytes, although this effect was milder. We have previously shown that in a macrophage under inflammatory conditions, Ang-2 stimulation enhances the production of inflammatory cytokines and chemokines [13,14], thus the effect observed in SSc monocytes seems to be potentiated by the inflammatory status of these monocytes [2,3]. Interestingly, the IL-6 and IL-8 levels are already higher in non-stimulated SSc monocytes and the inhibition of Tie2 signaling or the neutralization of Ang-2 abrogates the serum-induced IL-6 and IL-8 secretion. Therefore, the elevated serum levels of Ang-2 in SSc patients are, at least in part, responsible for the elevated levels of IL-6 and IL-8 cytokines in SSc pathology. IL-6 is elevated in circulation, affected skin, and bronchoalveolar lavage fluid from patients with SSc and can predict a worse outcome of the disease [2,7,21]. Regarding its functions, in vitro studies showed that IL-6 induces collagen production by SSc dermal fibroblasts. Importantly, a recent study has shown that the fibrotic role of IL-6 in not mediated by the transmembrane IL-6 receptor (IL-6R) signaling, but for a trans signaling initiated by the binding of IL-6 to the soluble IL-6 receptor (sIL-6R). This complex associates with the co-receptor gp130, which activates a Signal transducer and activator of transcription 3 (STAT3)-dependent signaling pathway, finally leading to collagen expression in an autocrine loop [22]. For this reason, we also analyzed the effect of Ang-2 on monocyte sIL-6R secretion. The secretion of sIL-6R by monocytes was really low (lower than 30 pg/mL) and it was not affected by Ang-2 stimulation (data not shown), suggesting that the fibrotic role of Ang-2 is mediated by the expression of IL-6 and not the soluble receptor involved in this signaling. In addition to the fibrotic role, IL-6 is also involved in the induction of TIMP-1 and in the increased frequency of Th17 cells [7]. Although IL-8 is also elevated in the serum of SSc patients, its role is not so well known. Evidence from literature suggests that IL-8 could be implicated in the recruitment of immune cells to the affected tissues like skin and lungs and, therefore, perpetuate the inflammatory processes [5,23].

Importantly, the serum levels of Ang-2 positively correlated with the skin involvement. This association is likely due to the elevated IL-6 levels, as this cytokine induces collagen deposition and also correlates with the modified Rodnan skin score (mRSS) [7]. In fact, IL-6 expression was elevated in the affected skin of dcSSc patients and was positively correlated with the skin *ANG2* levels (Figure S2A,B). Therefore, the Ang-2-induced IL-6 production might be involved in the fibrotic processes observed in the skin of SSc patients.

Some of the limitations of this manuscript are the small number of patients in our cohort and the lack of in vivo data. However, our work postulates Ang-2 as a potential therapeutic target for reducing monocyte-induced cytokine production. Due to the crucial role of Tie2 signaling on the maintenance of vascular integrity, and the pathological role of Ang-2 on vascular destabilization [9,24], the therapeutic use of anti-Ang2 antibodies may be more successful than the inhibition of the Tie2 signaling. However, further studies are needed to validate the therapeutic use of Ang-2 neutralization in SSc.

## 4. Materials and Methods

### 4.1. Patients and Controls

Patients’ blood from SSc patients and sex- and age-matched healthy controls was obtained from the University Medical Center Utrecht (Utrecht, The Netherlands) and the Maasstad Ziekenhuis Rotterdam (Rotterdam, The Netherlands). Cutaneous status was evaluated by the modified Rodnan skin score (mRSS). All patients provided informed written consent approved by the local institutional medical ethics review boards prior to inclusion in this study (Medical Research Ethics Committee (METC) of the University Medical Center Utrecht, METC numbers 12-466C (approved October 2, 2012) and 13/697 (approved April 30, 2014). Medical Research Ethics Committee (METC) of the Maastad Hospital, protocol L2014-66, approved November 20, 2014). Samples and clinical information were treated anonymously immediately after collection. All included patients fulfilled the ACR/EULAR 2013 classification criteria for SSc [25]. Demographics and clinical characteristics of the included patients and HCs are detailed in Table 1.

### 4.2. Monocyte Isolation

Peripheral blood mononuclear cells (PBMCs) from HC and SSc patients were isolated by Ficoll gradient (GE Healthcare, Chicago, IL, USA). Cells were processed for further isolation using magnetic beads and an autoMACS Pro Separator for CD14^+^ monocytes, according to the manufacturer’s instructions (Miltenyi Biotec, Bergisch Gladbach, Germany). Purity was assessed by flow cytometry and was routinely above 90%. Monocytes were cultured in RPMI-GlutaMAX (Thermo Fisher Scientific, Waltham, MA, USA) supplemented with 10% fetal bovine serum (FBS, Sigma, St. Louis, MO, USA) and 10,000 I.E penicillin-streptomycin (Thermo Fisher Scientific, Waltham, MA, USA).

### 4.3. Dermal Fibroblast Isolation and Culture

SSc dermal fibroblasts were isolated from 3–4 mm skin biopsies obtained from a clinically affected area. HC dermal fibroblasts were obtained from skin biopsies as resected material after cosmetic surgery. Dermal fibroblast isolation was performed using a whole skin dissociation kit (Miltenyi Biotec) following the manufacturer’s instructions and fibroblasts were routinely maintained in Dulbecco’s Modified Eagle Medium (Invitrogen, Carlsbad, CA, USA) supplemented with 10% FBS and 10,000 I.E penicillin-streptomycin. Cells were used for experiments between passages 3 and 5 and stimulations were performed after overnight starvation in medium containing 1% FBS.

### 4.4. Human Pulmonary Arterial Endothelial Cells Cell Culture

Human pulmonary arterial endothelial cells (HPAECs) (Thermo Fisher Scientific) were cultured in EBM-2 medium supplemented with EGM-2 bullet kit (both Lonza, Basel, Switzerland), 5% FBS and 10,000 I.E penicillin-streptomycin in 0.2% bovine skin gelatin-coated (Sigma Aldrich) culture vessels. HPAEC stimulation was performed after overnight starvation in EBM-2 basal medium with low serum for 24 h.

### 4.5. Cell Stimulation

Monocytes, dermal fibroblasts, and HPAECs were stimulated with LPS (100 ng/mL), R848 (1 µg/mL, both from Invivogen), or interferon alpha2a (IFN-α) (1000 U/mL, Cell Sciences) for 24 h and cells were lysed for mRNA expression analysis. Monocytes were also stimulated with Ang-2 (200 ng/mL) (from R&D Systems, Minneapolis, MN, USA) for 24 h and supernatants were collected for analysis of cytokine production. Alternatively, monocytes from HC were pre-incubated for 1 h at 37 °C with increasing concentrations of a Tie2 inhibitor (Selleck Chemicals, Houston, TX, USA) and further stimulated with serum of SSc patients (20% *v*/*v*) for 24 h. Finally, HC monocytes were pre-incubated for 1 h at 37 °C in the presence of Fc receptor (FcR) blocking (Miltenyi Biotec). SSc serum was also pre-incubated for 1 h at 37 °C in the presence of a human neutralizing anti-Ang2 antibody (Creative Biolabs, Shirley, NY, USA) or its respective isotype control (IgG2, R&D) and cultured with HC monocytes (20% *v*/*v*) for 24 h.

### 4.6. Flow Cytometry

PBMCs were stained with fixable viability dye (for dead cell exclusion, eBioscience, San Diego, CA, USA) and antibodies for CD14 APC (Miltenyi Biotec), CD11c PerCPCy5.5 (Biolegend, San Diego, CA, USA), HLA-DR FITC (BD Biosciences, Franklin Lakes, NJ, USA), CD16 V500 (BD Biosciences), Tie2 PE and its respective isotype control (R&D systems). Data were acquired on a BD FACSCanto II (BD Biosciences). After excluding debris, doublets, and dead cells, cell populations (see gating strategy in Table 2) were analyzed using the FlowJo software (Tree Star, Ashland, OR, USA). Results were expressed as percentage of positive cells.

### 4.7. RT-PCR and Quantitative (q)PCR

RNA from monocytes, dermal fibroblasts and HPAEC was isolated using the RNeasy micro Kit and RNase-Free DNase Set (Qiagen, Hilden, Germany). Total RNA was reverse-transcribed using an iScript cDNA Synthesis kit (Biorad, Hercules, CA, USA). PCR reactions were performed using SYBR green (Applied Biosystem, Foster City, CA, USA) with a StepOne Plus Real-Time PCR system (Applied Biosystems). cDNA was amplified using specific primers (all from Integrated DNA Technologies, Inc. (IDT), Coralville, IA, USA, see Table 3). Relative levels of gene expression were normalized to *GAPDH* housekeeping gene. The relative quantity of mRNA was calculated using the formula 2^−ΔCt^ × 1000.

### 4.8. Ang-2 Expression from Profiling Data

*ANG2* and *IL6* gene expression was retrieved from array profiling data available on the Gene Expression Omnibus (GEO–NCBI) (GSE95065).

### 4.9. Measurement of Cytokine Production

Serum Ang-2 levels were determined by ELISA (R&D systems) as well as IL-6 and IL-8 secretion in cell-free supernatants (PelKine Compact™ ELISA kits, Sanquin Reagents, Amsterdam, The Netherlands).

### 4.10. Statistical Analyses

Statistical analysis was performed using Windows GraphPad Prism 8 (www.graphpad.com, GraphPad Software, Inc., San Diego, CA, USA). Potential differences between experimental groups were analyzed by non-parametric, Kruskal–Wallis test and Friedman test, or parametric ANOVA test when appropriate. For correlations with disease parameters, Spearman’s rho was used. *p*-values < 0.05 were considered statistically significant.

## Figures and Tables

**Table 1:** **Characteristics of the SSc patients and controls.** Data are presented as the median (interquartile range) or number (percentage). ANA: antinuclear antibodies; ACA: anticentromere antibodies; Scl70: antitopoisomerase I antibodies; mRSS: modified Rodnan skin score; FCV: forced vital capacity; ILD: interstitial lung disease; DMARDs: disease-modifying antirheumatic drugs; N.D.: not determined.

Patient Characteristics	ELISA Angiopoietins		Dermal Fibroblasts		Functional Assays	
	HC (*n* = 20)	SSc (*n* = 27)	HC (*n* = 6)	SSc (*n* = 5)	HC (*n* = 24)	SSc (*n* = 27)
Age (years)	44 (37–50)	64 (49–68)	40 (40–52)	42 (36–45)	50 (42–57)	53 (42–66)
Female: *n* (%)	17 (85)	21 (78)	6 (100)	6 (60)	18 (75)	19 (70)
Disease duration (years)		9 (2–14)		2 (1–3)		8 (3–15)
Limited cutaneous SSc		15 (55)		1 (20)		11 (40)
Diffuse cutaneous SSc		12 (45)		4 (80)		16 (60)
ANA positive: *n* (%)		26 (96)		4 (80)		26 (96)
ACA positive: *n* (%)		9 (33)		0 (0)		7 (26)
Scl70 positive: *n* (%)		8 (27)		1 (20)		13 (48)
mRSS		4 (2–9)		7 (7–8)		7 (4–13)
FVC		104 (90–119)		N.D.		91 (83–98)
ILD: *n* (%)		7 (26)		1 (20)		6 (22)
DMARDs: *n* (%)		18 (66)		4 (80)		15 (55)
Biologicals: *n* (%)		0 (0)		0 (0)		1 (4)

**Table 2:** Phenotypic strategy used to identify the different cell populations.

Cell Population	Gating Strategy
Monocytes	HLA-DR^+^ CD11c^+^ CD14^+^
Classical monocytes	HLA-DR^+^ CD11c^+^ CD14^+^ CD16^−^
Intermediate monocytes	HLA-DR^+^ CD11c^+^ CD14^+^ CD16^+^
Non-classical monocytes	HLA-DR^+^ CD11c^+^ CD14^−^ CD16^+^

**Table 3:** List of PCR primers.

Gene	Primer Forward	Primer Reverse
*ANG2*	5′TCAGTGGCTAATGAAGCTTGAGA3′	5′CCGCTGTTTGGTTCAACAGG3′
*GAPDH*	5′GCCAGCCGAGCCACATC3′	5′TGACCAGGCGCCCAATAC3′

## Supplementary Materials

The following are available online at https://www.mdpi.com/1422-0067/21/24/9544/s1. Figure S1: Ang-2 levels are elevated in both SSc subsets, Figure S2: IL-6 expression is elevated in the skin of SSc patients and positively correlates with Ang-2 levels.
