# Supplementary material for: Angiopoietin-2 Promotes Inflammatory Activation in Monocytes of Systemic Sclerosis Patients
Source: Int J Mol Sci. 2020 Dec 15;21(24):9544. doi: 10.3390/ijms21249544 (PMC7765391; doi:10.3390/ijms21249544)
Supplement: Supplementary file 1 [file ijms-21-09544-s001.pdf]

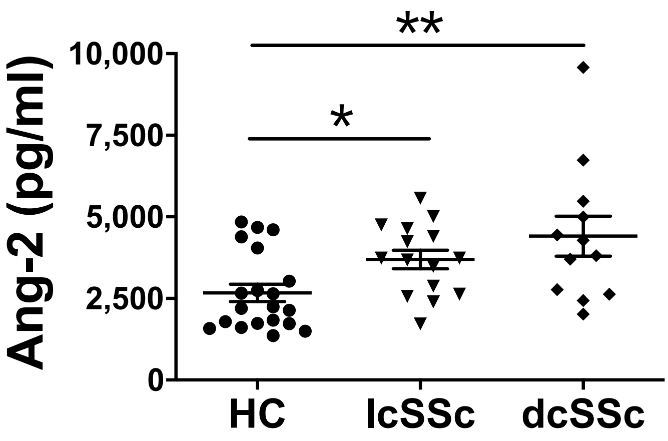

Supplementary Figure S1. Ang-2 levels are elevated in both SSc subsets. Ang-2 levels in serum from HC (n = 20), lcSSc (n = 15) and dcSSc patients (n=12). Mean and SEM are shown. * $p < 0.05$ and ** $p < 0.01$.

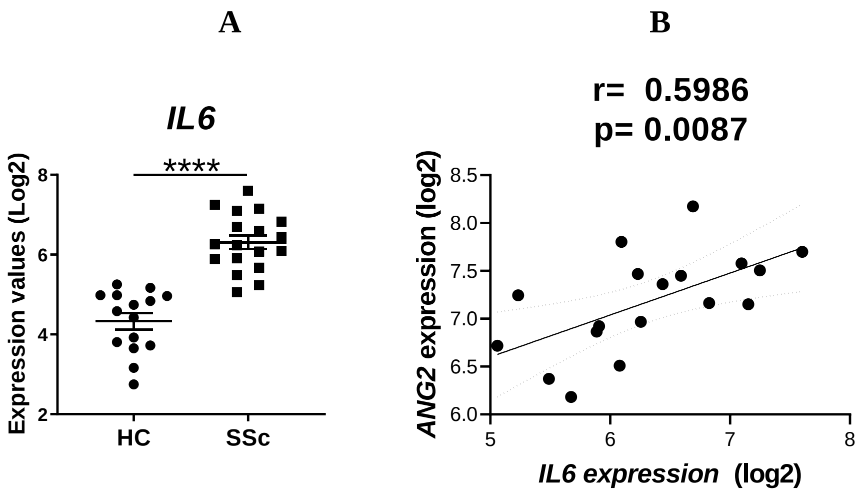

Supplementary Figure S2. **IL-6 expression is elevated in the skin of SSc patients and positively correlates with Ang-2 levels. (A)** *IL6* expression in the skin of HC (n = 15) and affected skin of SSc patients (n = 18). **(B)** Correlation analysis of *ANG2* and *IL6* expression in the affected skin of SSc patients. **** $p < 0.0001$.
